# On-demand chlorine dioxide solution enhances odontoblast differentiation through desulfation of cell surface heparan sulfate proteoglycan and subsequent activation of canonical Wnt signaling

**DOI:** 10.3389/fcell.2023.1271455

**Published:** 2023-10-26

**Authors:** Toshihiro Inubushi, Nag Priyanka, Masakatsu Watanabe, Yusuke Takahashi, Shinnosuke Kusano, Hiroshi Kurosaka, Silvana Papagerakis, Petros Papagerakis, Mikako Hayashi, Takashi Yamashiro

**Affiliations:** ^1^ Department of Orthodontics and Dentofacial Orthopedics, Graduate School of Dentistry, Osaka University, Suita, Japan; ^2^ Department of Restorative Dentistry and Endodontology, Graduate School of Dentistry, Osaka University, Suita, Japan; ^3^ Department of Surgery, College of Medicine, University of Saskatchewan, Saskatoon, SK, Canada; ^4^ Department of Anatomy, Physiology and Pharmacology, College of Medicine, University of Saskatchewan, Saskatoon, SK, Canada; ^5^ College of Dentistry, University of Saskatchewan, Saskatoon, SK, Canada; ^6^ Department of Otolaryngology-Head and Neck Surgery, School of Medicine, University of Michigan, Ann Arbor, MI, United States

**Keywords:** heparan sulfate proteoglycans, chlorinated oxidant, MA-T, DSPP, DMP1

## Abstract

Heparan sulfate proteoglycans (HSPGs) surround the surface of odontoblasts, and their modification affects their affinity for Wnt ligands. This study proposes applying Matching Transformation System^®^ (MA-T), a novel chlorinated oxidant, to enhance dentinogenesis. MA-T treatment in odontoblasts decreased sulfation of HSPG and upregulated the expression of *dentin sialophosphoprotein* (*Dspp*) and *Dentin Matrix Protein 1* (*Dmp1*) via activation of canonical Wnt signaling *in vitro*. *Ex vivo* application of MA-T also enhanced dentin matrix formation in developing tooth explants. Reanalysis of a public single-cell RNA-seq dataset revealed significant Wnt activity in the odontoblast population, with enrichment for *Wnt10a* and *Wnt6*. Silencing assays showed that *Wnt10a* and *Wnt6* were redundant in inducing *Dspp* and *Dmp1* mRNA expression. These Wnt ligands’ expression was upregulated by MA-T treatment, and TCF/LEF binding sites are present in their promoters. Furthermore, the Wnt inhibitors Notum and Dkk1 were enriched in odontoblasts, and their expression was also upregulated by MA-T treatment, together suggesting autonomous maintenance of Wnt signaling in odontoblasts. This study provides evidence that MA-T activates dentinogenesis by modifying HSPG and through subsequent activation of Wnt signaling.

## Introduction

Dentin is a calcified product of odontoblasts and has a matrix composition similar to bone but is characterized by the formation of dentin tubules aligned in the matrix and the absence of remodeling. In contrast to osteoblasts, odontoblasts are characterized by cell polarity and odontoblasts specifically express dentin-specific extracellular matrix proteins, *dentin sialophosphoprotein* (*Dspp*), and *dentin matrix protein 1 (Dmp1)* (D'Souza et al., 1997). Null mutants of these protein genes show specific defective expression in dentin but not in bone. The absence of polarity cells on the surface of mutant dentin (Sreenath et al., 2003; Ye et al., 2004) suggests an essential and specific role for *Dspp* and *Dmp1* in dentin matrix formation.

Among several signaling pathways, the canonical Wnt pathway is involved in dentin formation, as evidenced by Wnt-reporter (*TOPGAL*) and *Axin2* reporter activity in developing tooth odontoblasts (Lohi et al., 2010; Suomalainen and Thesleff, 2010). Furthermore, Wnt signaling is inhibited in Dickkopf-related protein 1 (Dkk1) overexpressing mice, and dentin formation is impaired, demonstrating the important role played by Wnt signaling (Han et al., 2011). Conversely, the treatment with LiCl, an activator of canonical Wnt signaling through inhibition of glycogen synthase kinase-3 (GSK-3) (Hayano et al., 2012), upregulated Dspp expression *in vitro* and stimulated the dentin repair *in vivo* (Ishimoto et al., 2015).

Both the *Dspp* and *Dmp1* promoters have TCF/LEF binding sites (Nakatomi et al., 2013). In the developing teeth, *Wnt10a* is specifically expressed in the odontoblast *in vivo*, and overexpression of *Wnt10a* increased *Dspp* and *Dmp1* expression in odontoblast cells (Yamashiro et al., 2007). WNT6 has also been associated with increased mineral matrix formation in human dental pulp cells and increased Dspp and Dmp1 expression (Li et al., 2014). However, *Wnt10*a-deficient mice have fewer dentin defects (Yang et al., 2015), and no dental phenotype has been reported in rodent *Wnt6-*deficient mice (Wang Q. et al., 2013), suggesting that there may be some redundancy between *Wnt10a* and *Wnt6* or that these other Wnt ligands are involved in dentin formation. Thus, the Wnt signaling network in dentin formation is complex, and a comprehensive analysis is needed to clarify the role of Wnt signaling.

Interestingly, Wnt activity is influenced by the surrounding microenvironment, particularly the sulfation status of cell surface heparan sulfate proteoglycan (HSPG) (Ai et al., 2003). In odontoblasts, double knockout of the extracellular endosulfatases *sulfatase* (*Sulf*)1 and *Sulf2* results in dentin dysplasia due to the downregulation of the canonical Wnt signaling pathway (Hayano et al., 2012). These enzymes remove sulfate residues from HSPGs, resulting in reduced affinity of cell surface HSPGs for WNT10a, allowing WNT ligands to translocate to Wnt receptors and activate Wnt signaling (Hayano et al., 2012). Such a process can be mimicked by the pharmaceutical application of sodium chlorate *in vitro* (Ishimoto et al., 2015). Sodium chlorate is a potent oxidant, and its application *in vitro* removes the sulfated residues of cell surface HSPGs (Ai et al., 2003) that subsequently activate the Wnt signaling and upregulate the *Dspp* expression in the odontoblast cell line (Hayano et al., 2012). However, it generates toxic chlorine gas upon contact with oxidizing substances or by self-decomposition. In addition, the radicals in this reaction are highly reactive, making their clinical use as dentin regenerative agents difficult.

Matching Transformation System^®^ (MA-T) was developed as a next-generation chlorine disinfectant with high safety and anti-pathogen activity (Acenet Inc., Japan). MA-T contains sodium chlorite with cationic detergents acting as Lewis acids to catalyze chlorine dioxide generation in a buffer that stabilizes the solution at a neutral pH (Suomalainen and Thesleff, 2010; Shibata and Konishi, 2020; Shibata et al., 2021). In the presence of a reactive target, active species are consumed, and the chemical equilibrium equation allows the production of new active species only in the amount consumed and never produces chlorine dioxide gas (Shibata and Konishi, 2020; Shibata et al., 2021). Notably, no chlorine dioxide has been detected in MA-T during storage. The safety of MA-T has been demonstrated in animal and human safety and toxicity studies (Shibata and Konishi, 2020), and its antimicrobial and antiviral activities have already been demonstrated (Shibata et al., 2021; Noguchi et al., 2022a; Urakawa et al., 2022; Ono-Minagi et al., 2023a; Yamamoto et al., 2023). However, its oxidative effects on the cell surface HSPG have not been evaluated. This study proposes a novel approach to odontogenic differentiation using MA-T, mimicking the sugar chain modification on odontoblasts in nature. We also reanalyze the publicly available single-cell RNA sequencing (scRNA-seq) data to demonstrate the critical role of Wnt signaling in dentinogenesis and show how MA-T regulates odontoblast differentiation by activating Wnt signaling.

## Results

### Wnt activity in the developing tooth

**FIGURE 1:**
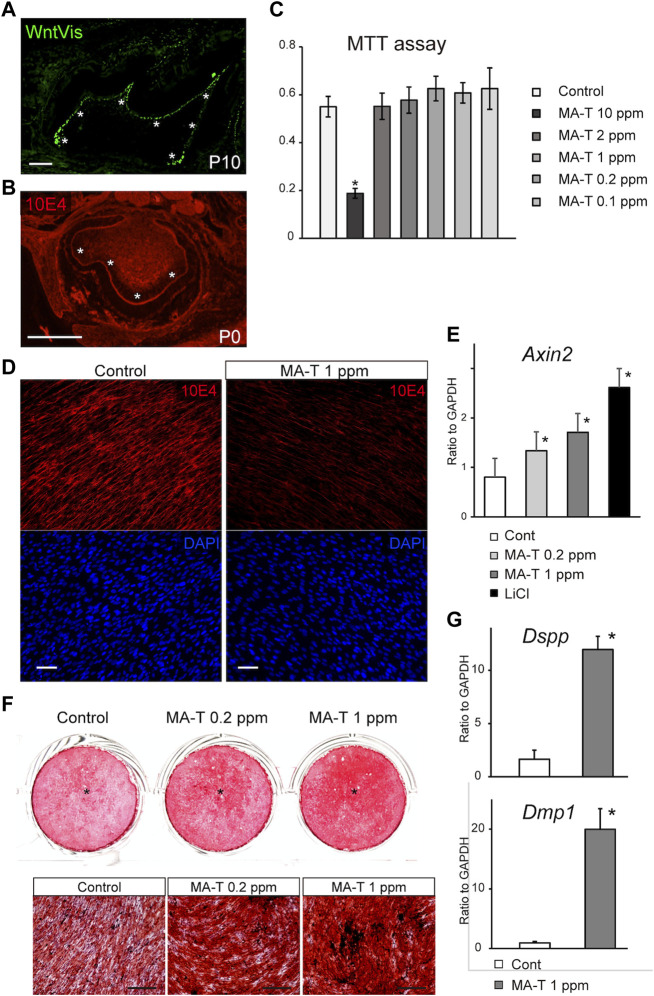
MA-T treatment and Wnt signaling in odontoblast cells. **(A)** WntVis reporter activity in developing molars at p10. Signals are specifically intense in odontoblasts (arrowheads). **(B)** Immunofluorescence staining for 10E4 in developing molars at P0. Immunofluorescence staining for 10E4. 10E4 immunoreactivity was evident in the basement membrane and pulp cells but was absent in the odontoblasts (arrowhead). **(C)** MTT assay for cell viability after exposure to 0.1–10 ppm MA-T. MA-T exposure of less than 0.2 ppm did not affect cell viability compared to the control. Means ± SD (*n* = 5) are shown as horizontal bars. *p* values were determined by one way ANOVA. *p < 0.01. **(D)** 10E4 immunoreactivity of odontoblasts after exposure to MA-T. The 10E4 immunoreactivity was reduced by 1.0 ppm MA-T. **(E)** A qPCR analysis of the odontoblasts 3 days after treatment with MA-T or LiCl. MA-T treatment upregulated Axin2 mRNA expression, as did LiCl. Means ± SD (*n* = 3) are shown as horizontal bars. *p* values were determined by one way ANOVA. *p < 0.01 **(F)** Alizarin red S staining of odontoblast cells 14 days after treatment with 0.2 and 1.0 ppm MA-T. The MA-T treatment increased the formation of calcified nodules. **(G)** A qPCR analysis of odontoblasts 14 days after treatment with MA-T. MA-T treatment significantly upregulated both Dspp and Dmp1 mRNA expression. Means ± SD (*n* = 3) are shown as horizontal bars. *p* values were determined by unpaired Student’s t-test. *p < 0.01. Scale bars: 200 μm in **(A, B, F)**; 50 μm in D.

A previous study using TOPGAL reporter mice provided evidence for canonical Wnt signaling (Lohi et al., 2010; Suomalainen and Thesleff, 2010). We first evaluated this activity more extensively using WntVis reporter mice at P7.0 (Takemoto et al., 2016). Signals were intense in the odontoblast layer (asterisks in Figure 1A). Weak Signals were detected at the alveolar bone surface and the periodontal ligament (Figure 1A).

### Desulfation in the developing tooth

10E4 recognizes the sulfated HSPG, and we have previously shown that HSPG is desulfated in mature odontoblasts (Hayano et al., 2012). In developing molars at P0, 10E4 immunoreactivity was absent from the odontoblast layers (Figure 1B), suggesting that cell surface HSPG was desulfated with odontoblast differentiation. Tmem2-CKO.

### Cell viability in response to MA-T

The cell viability of primary mouse dental papilla mesenchymal cells (mDP cells) after exposure to 0.1–10 ppm MA-T for 48 h was measured using the MTT (3-(4,5-dimethyl-2-thiazolyl)-2,5-diphenyltetrazolium bromide) assay. The viability of mDP cells was significantly reduced by MA-T treatment at 10 ppm. MA-T exposure at less than 0.2 ppm did not affect cell viability compared to the control (Figure 1C).

### Desulfation of cell surface HSPG by MA-T

Sodium chlorate is a potent oxidant and a reversible inhibitor of glycosaminoglycan sulfation. Treatment with chlorate significantly reduced the binding of the 10E4 antibody to odontoblasts (Hayano et al., 2012). The main active ingredient of MA-T is chlorine dioxide which has oxidizing properties. However, neither MA-T nor chlorine dioxide has been tested for effectiveness in the desulfating of HSPG in odontoblasts.

10E4 immunoreactivity was evident in odontoblasts, while 72 h MA-T treatment resulted in a marked reduction in the cell-surface 10E4 immunoreactivity in odontoblast mDP cells (Figure 1D), indicating that MA-T desulfated HSPG on odontoblasts, as well as sodium chlorate.

### Activation of Wnt signaling and odontoblast differentiation by MA-T

MA-T treatment also upregulated the mRNA expression of Axin2, a canonical Wnt signaling marker, 3 days after MA-T application (Figure 1E).

We then cultured odontoblast cell lines until they formed a mineralized matrix. MA-T treatment increased mineralized matrix deposition as assessed by alizarin red S incorporation 14 days after the start of culture (Figure 1F). MA-T also upregulated the expression of *Dspp* and *Dmp1* (Figure 1G).

### Enhancement of the dentin matrix formation by MA-T

**FIGURE 2:**
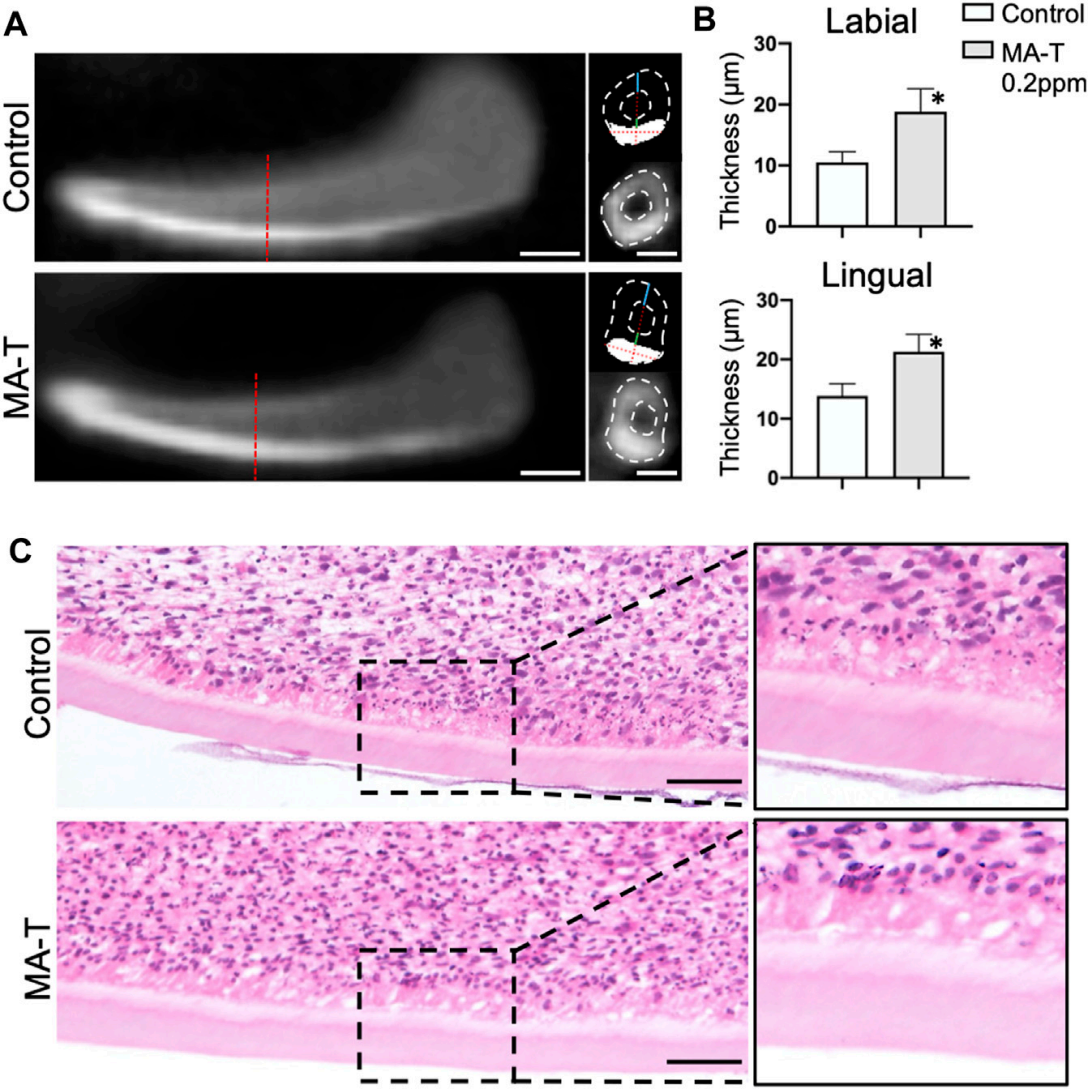
**(A)** Sagittal and transverse micro-CT reconstructed images of lower incisor explants incubated with or without MA-T for 10 days. **(B)** Dentin thickness at the lingual and labial side of the incisor were measured. Dentin thickness was increased by the MA-T treatment. Means ± SD (*n* = 3) are shown as horizontal bars. *p* values were determined by unpaired Student’s t-test. *p < 0.05. **(C)** Sagittal histologic images of lower incisors treated with or without MA-T. Scale bars: 200 μm in A; 100 μm in C.

To further analyze the influence of MA-T on dentin matrix formation, the growing incisors at E16.0 were cultured with treatment of 1.0 ppm of MA-T for 10 days. Dentin matrix formation was evaluated by micro-CT and histologic evaluation. The enamel was highly calcified in the CT images (Figure 2A), whereas the dentin appeared comparatively less calcified. To determine the thickness of the dentin matrices, we first measured the thickness of calcified tissue on the labial aspect of the incisors. The thickness of the enamel was then measured at the designated measurement site using signal threshold adjustments. Finally, the thickness of dentin was calculated as the difference between the two. The results showed that MA-T increased the thickness of dentin (Figure 2B). Histologic images showed the formation of dentin and an increase in the size of dentin matrices due to MA-T (Figure 2C).

### Involvement of Wnt signaling in dentinogenesis in reanalysis of public sc-RNA data

To comprehensively understand the Wnt signaling in odontoblast differentiation, we reanalyzed the public GSE146855 scRNA-seq dataset on the previously analyzed isolated mouse incisors (Chiba et al., 2020).

**FIGURE 3:**
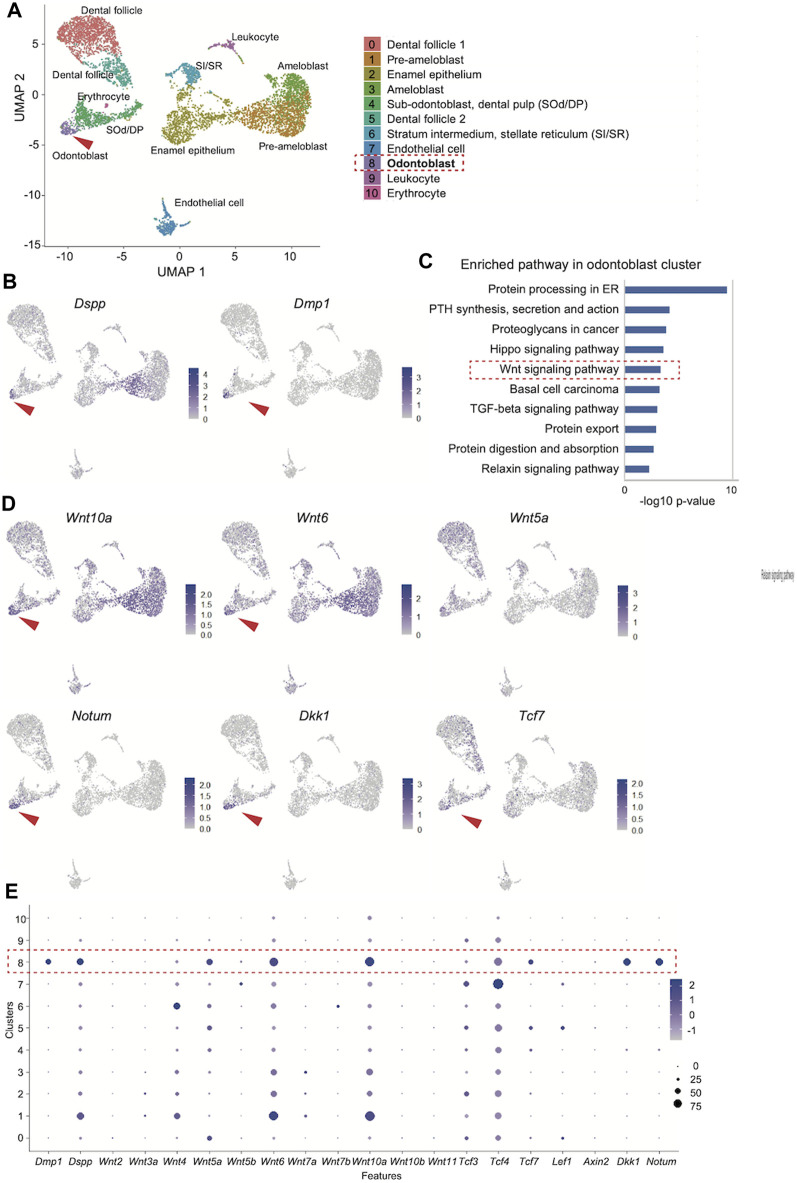
Reanalysis of the public scRNA-seq dataset (GSE146855) of the isolated mouse incisors. **(A)** UMAP visualization of transcriptionally distinct cell populations in cells from isolated mouse incisors. All cells are colored according to their cellular identity. **(B)** Expression of Dspp and Dmp1 projected onto the UMAP plot of mouse incisors. **(C)** KEGG pathway analysis of the 300 DEGs in the odontoblast cluster revealed that “Wnt signaling pathway” was highly enriched (-log10 *p* values = 3.33). **(D)** Expression of enriched “Wnt signaling pathway"-related genes on UMAP plot. **(E)** Dot plot visualization of Wnt-related genes in each cluster. The red dotted square indicates the odontoblast cluster. Dot size reflects the percentage of cells in a cluster expressing each gene; dot color reflects expression level.

We obtained 6,260 single-cell transcriptome data, and uniform manifold approximation and projection (UMAP) clustering identified ten cell population clusters of odontoblast, sub-odontoblast and dental pulp, dental follicle 1 and 2, ameloblast, pre-ameloblast, quiescent-IEE, non-ameloblast, leukocyte, erythrocyte, endothelial cell (Figure 3A). The annotation of these cell types for these clusters was based on the expression of the specific markers in different clusters on the UMAP plot (Supplementary Figures S1A, B). In addition, the expression of *Dspp* and *Dmp1* was projected onto the UMAP plot, which showed that the distribution of the Dmp1-expressing cell population was similar to that of Dspp in the odontoblast clusters (Figure 3B).

Next, the odontoblast cluster was characterized. The FindAllMarkers function in Seurat identified the 524 differentially expressed genes in the odontoblast cluster (0.05 > *p*-value adjusted). KEGG pathway analysis of the top 300 up- and downregulated genes in the odontoblast cluster revealed that the “Wnt signaling pathway” was highly enriched (-log10 *p* values = 3.33, Figure 3C). In this pathway, enriched DEGs were Bambi, Dkk1, Lgr6, Nkd1, Notum, Serpinf1, Tcf7, Wnt10a, Wnt5a, Wnt6. Among these genes, feature analysis revealed that the distribution of Dspp and Dmp1 was uniquely overlapped with that of Wnt6, Wnt10, Dkk1, and Notum (Figure 3D). The distribution of Tcf7 expression also overlapped with Dspp and Dmp. Tcf7 was more specifically distributed in the odontoblast cluster than Lef1, Tcf3, or Tcf4 (Figure 3D and Supplementary Figure S2). Bambi and Serpinf1 were highly expressed in odontoblasts, but their expression was rather non-specific. Wnt5a expression was also non-specific among odontoblasts, pre-odontoblasts, and dental mesenchymal cells (Supplementary Figure S2).

DotPlot showed that the Dmp1 expression is more specific than Dspp. Dotblot also confirmed that Wnt6, Wnt10a, and Wnt5a were more expressed in the odontoblast cluster. Tcf7 is more specific in the odontoblast cluster than Tcf3 and Tcf4 (Figure 3E).

### Functional redundancy of Wnt10a and Wnt6

Previous studies have shown that both Wnt10a and Wnt6 have anabolic effects on odontoblast differentiation. The scRNA-seq reanalysis showed the specific overlap of *Wnt6* and *Wnt10a* expressing cells among odontoblast clusters. Therefore, we hypothesized that they have some functional redundancy.

**FIGURE 4:**
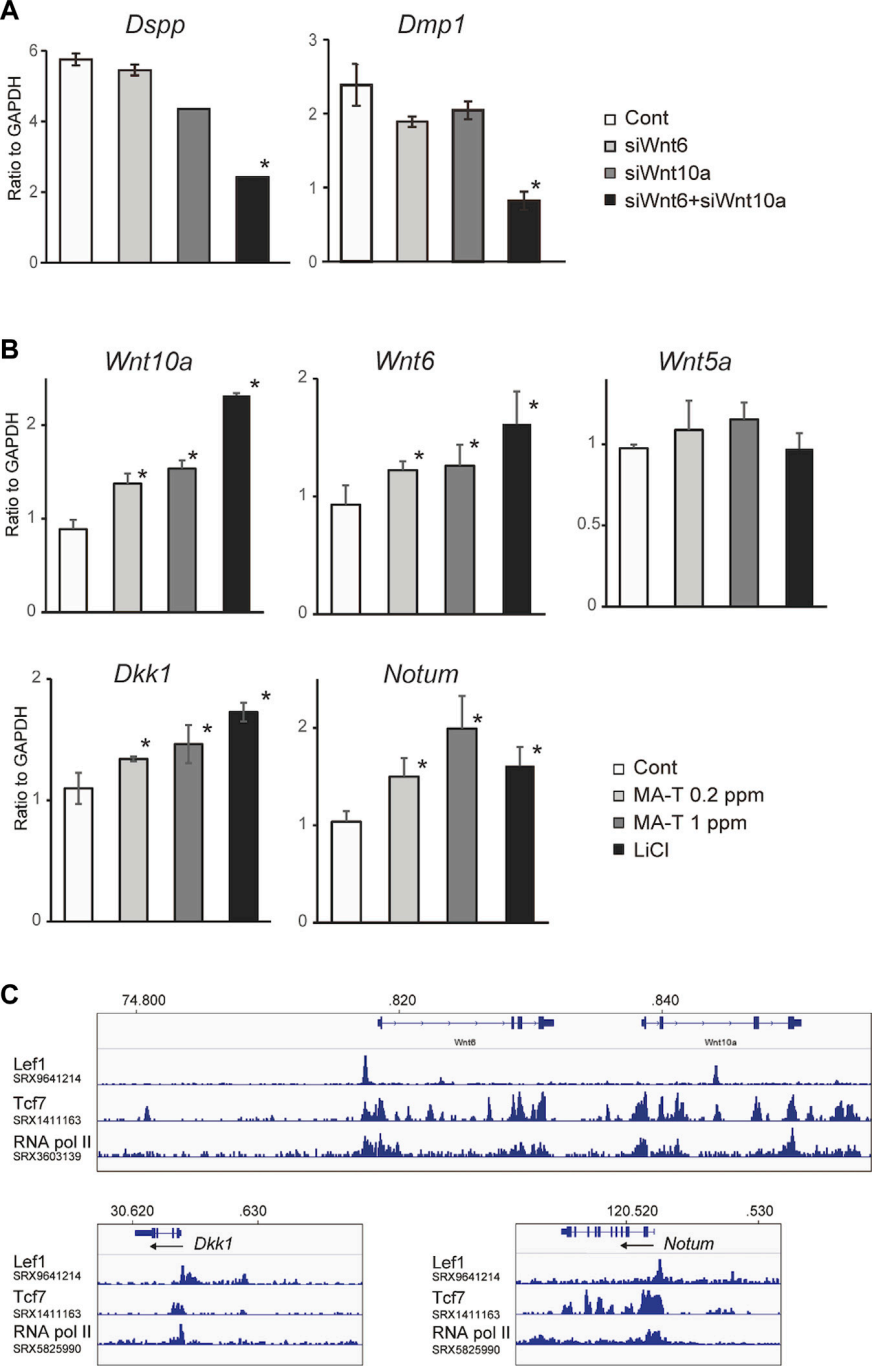
**(A)** A qPCR analysis of the odontoblast after inhibition of Wnt10a and Wnt6. The combination of Wnt10a siRNA with Wnt6 siRNA significantly downregulated the expression of Dspp and Dmp1, respectively, compared with the single treatment. Means ± SD (*n* = 3) are shown as horizontal bars. *p* values were determined by one way ANOVA. *p < 0.01. **(B)** Expression of “odontoblast cluster”-specific Wnt-related genes after treatment with MA-T and LiCl. A qPCR analysis showed that the expression of *Wnt10a, Wnt6, Dkk1,* and *Notum* was upregulated by either. Means ± SD (*n* = 3) are shown as horizontal bars. *p* values were determined by one way ANOVA. *p < 0.01. **(C)** In silico ChIP analysis of TCF/LEF binding in *Wnt10a, Wnt6, Dkk1,* and *Notum.*

The combination of *Wnt*10a siRNA with *Wnt*6 siRNA showed a more remarkable reduction in the expression of *Dspp* and *Dmp1*, respectively, compared to the single treatment (Figure 4A), suggesting that there might be functional redundancy between Wnt10a and Wnt6 in the induction of odontoblast differentiation.

### Regulation of Wnt6, Wnt10a, Notum and Dkk1 expression by Wnt activation or MA-T treatment

Wnt signaling is activated in odontoblasts as shown by WntBis reporter mice (Figure 1A). In our scRNA-seq, both Wnt ligands and Wnt inhibitory genes are specifically activated in odontoblasts. Thus, we speculate that Wnt activity is maintained in the odontoblast by both direct activation and inhibition of Wnt signaling in an autocrine manner.

Our *in vitro* studies showed that MA-T treatment at 0.2 ppm and 1.0 ppm and LiCl treatment upregulated the mRNA expression of Wnt10a and Wnt6 but not Wnt5a in odontoblasts (Figure 4B). On the other hand, Wnt inhibitors of both Dkk1 and Notum were also activated by MA-T and LiCl treatment (Figure 4B). Thus, these results reveal the existence of a positive and negative feedback loop in which Wnt signaling in odontoblasts is maintained between Wnt ligands and Wnt inhibitors.

To evaluate the possible direct control of Wnt6, Wnt10a, Notum, and Dkk1 by Wnt signaling, we investigated whether TCF/LEF binding sites exist around the promoter region of *Wnt10a, Wnt6, Dkk1,* and *Notum* by analyzing the public ChIP-seq data (SRX9641211, SRX1411163, SRX360319, SRX5825990) using *Lef1, Tcf7*, and RNA polymerase II. In silico ChIP analysis was performed using the ChIP-Atlas 23.

ChIP-Atlas analysis showed that Tcf7 occupies the proximal promoter region of *Wnt6, Wnt10a, Notum,* and *Dkk1* with *RNA pol II* (Figure 4C). Lef1 also bind to binds to the promoters of Wnt6, Notum, and Dkk1 (Figure 4C).

### Cell viability and induction of odontoblast differentiation by sodium chlorite

MA-T contains sodium chlorite, but cationic detergents acting as Lewis acids allow sodium chlorite to be converted to chlorine dioxide only in the presence of reactive targets (Shibata and Konishi, 2020).

We evaluated the cell viability of the mDP cells in response to sodium chlorite treatment by MTT assay and the influence on odontoblast differentiation. The viability of mDP cells was significantly reduced by sodium chlorite treatment at 10 ppm. However, sodium chlorite exposure at less than 1.0 ppm did not affect the cell viability in contrast to the control (Supplementary Figure S3A). On the other hand, sodium chlorite upregulated the expression of both *Dmp1* and *Dspp* expression at the dose of 100 ppm. Such upregulation was not evident at the dose of less than 2.0 ppm (Supplementary Figures 3B, C), suggesting that sodium chlorate could promote the odontoblast differentiation only at a toxic dose to mDP cells.

## Discussion

Our previous *in vitro* study showed that sodium chlorate enhances *Dspp* and *Dmp1* expression with activation of canonical Wnt signaling by modifying the sulfated state of cell surface odontoblast HSPG 7. Such findings lead us to the potential application of sodium chlorate in dentin regeneration 8. However, sodium chlorate is a strong oxidant. It is commonly used as a chlorine dioxide-generating disinfectant, and conventional chlorine-based disinfectants release toxic chlorine dioxide gas and are highly reactive due to free radical generation (Lewis Ivey and Miller, 2013). Therefore, we have been reluctant to use this agent for clinical use. MA-T is being developed as a next-generation chlorine dioxide-generating disinfectant (Shibata and Konishi, 2020; Shibata et al., 2021). Conventional chlorine-based disinfectants release toxic chlorine dioxide gas and are highly reactive (Lewis Ivey and Miller, 2013). Specifically, the modified catalyst in MA-T controls the generation of aqueous chlorine dioxide radicals in a buffer stabilizing the solution (Shibata et al., 2021). Oral safety studies have shown that MA-T can be safely used at a minimum of 100 ppm without affecting the high anti-pathogen activity (Shibata and Konishi, 2020; Shibata et al., 2021). However, the effect of MA-T on dentinogenesis remains to be elucidated. This study provided the first *in vitro* evidence that MA-T modulates the sulfated state of the cell surface HSPG on the odontoblasts (Figure 1D) and subsequently activates odontoblast differentiation via activation of canonical Wnt signaling *in vitro* (Figure 1E). In addition, our study provided the first *ex vivo* evidence that MA-T promotes dentinogenesis (Figure 4). These results suggest that MA-T has the potential to be a safe and effective alternative to sodium chlorate for promoting dentin regeneration.

The present R26-WntVis reporter signals support that the canonical Wnt signaling is specifically activated in the *Dspp-* and *Dmp1-*expressing odontoblast layer (Figure 1A), as well as previous findings using TOPGAL reporter signals (Suomalainen and Thesleff, 2010). However, how Wnt signaling is important among different signaling pathways and which Wnt genes are involved in dentinogenesis remain to be elucidated. Furthermore, because many Wnt-related genes exist in the human and mouse genomes, it is difficult to determine which Wnt ligands or TCF/LEF are involved in odontoblast differentiation. Therefore, a comprehensive understanding of the role of Wnt signaling in odontoblasts was needed. The present reanalysis of the public scRNA-seq dataset (Chiba et al., 2020) clearly showed that “Wnt signaling pathway” was enriched in the next lowest *p*-value next to the “Hippo signaling pathway” in the segregated odontoblast cluster. KEGG analysis further picked up the genes of *Bambi, Dkk1, Lgr6, Nkd1, Notum, Serpinf1, Tcf7, Wnt10a, Wnt5a* and *Wnt6*, as Wnt-related genes in this cluster. Indeed, the expression of these genes on the UMAP projection (feature plot) also supported that these genes were highly expressed in the odontoblast cluster (Figure 2D). Among these genes, the feature plot also showed that the expression of *Wnt10a, Wnt6, Dkk1, Notum,* and *Tcf7* was overlapped with the expression of *Dspp* and *Dmp1*. Thus, reanalysis of scRNAseq data confirmed that Wnt signaling is specifically activated during odontoblast differentiation and also suggested suggests that the Wnt ligands Wnt10a and Wnt6 and the Wnt inhibitors Dkk1 and Notum colocalize specifically in dentin-forming odontoblasts.

Interestingly, LiCl and MA-T were found to upregulate the expression of the Wnt ligands Wnt10a and Wnt6 and the Wnt inhibitors Dkk1 and Notum (Figure 3B). These results suggest that both Wnt activators and inhibitors regulate Wnt signaling through both positive and negative feedback loops. Our analysis of public ChIP-seq data showed that TCF binding was closely colocalized with RNA Pol II binding in all of these genes (Figure 3C), suggesting that these Wnt10a, Wnt6, Dkk1, and Notum are directly regulated by canonical Wnt signaling. Thus, our results and analysis of public datasets demonstrated that canonical Wnt signaling would be tightly maintained by a feedback loop of both Wnt activity and repression during odontoblast differentiation.

Previous studies have shown that both *Wnt10a* and *Wnt6* upregulate the expression of *Dspp* and *Dmp1 in vitro* (Yamashiro et al., 2007; Wang et al., 2010; Hayano et al., 2012; Li et al., 2014; Zhang et al., 2014). However, the null mutant of either *Wnt10a* or *Wnt6* does not show a severe dentin defect. Indeed, *Wnt10a* deficiency disrupted the dentin formation only on the lingual side but did not disrupt odontoblast differentiation (Yang et al., 2015). The present reanalysis of scRNA-seq data showed that *Wnt6* and *Wnt10a* showed a striking overlap between them. Therefore, their functional redundancy likely explains why complete dentinogenesis was not disrupted in any *Wnt6* or *Wnt10a* null mutant. *Wnt10a* and *Wnt6* may be specifically and synergistically involved in odontoblast differentiation. In the future, it is expected that a double knockout of Wnt10a and Wnt6 is expected to reveal redundant functions of Wnt10a and Wnt6.

In our analysis, Notum was enriched in the odontoblast cluster with a similar distribution of *Dspp* and *Dmp1. Notum* suppresses the Wnt activity (Kakugawa et al., 2015), and its null mutant showed dentin dysplasia with a porous dentin matrix formation with constricted dental pulp (Vogel et al., 2016). Dkk1 is also a Wnt inhibitor and was identified in this cluster. Overexpression of Dkk1 under the Col1a1 promoter results in reduced dentinogenesis, more immature odontoblasts, and fewer dentin tubules (Han et al., 2011). In contrast to bone, there is no remodeling of the mineralized matrix and the balance between pulp and dentin volume is maintained throughout life. Dentinogenesis is likely to be tightly regulated, at least in part, by the balance between Wnt activation and inhibition.

The MTT assay showed little difference in cytotoxicity between sodium chlorite and MA-T. On the other hand, MA-T increased Dspp expression at non-toxic concentrations, whereas sodium chlorite increased Dspp expression only at high toxic concentrations. Therefore, the present experiments confirmed for the first time that sodium chlorite promotes odontoblast differentiation, but its high cytotoxicity makes its clinical application difficult. MA-T enhanced Dspp expression at much lower concentrations than sodium chlorite. MA-T contains a cationic detergent that acts as a Lewis acid, efficiently generating ClO_2_ while maintaining equilibrium and not producing toxic chlorine dioxide gas.

Disinfectants can induce cellular senescence, so care should be taken to avoid the induction of cellular senescence associated with the administration of MA-T (Al-Azab et al., 2022; Lyamina et al., 2023). In our *in vitro* study, MA-T did not induce cellular senescence under the conditions of this experiment. However, when studying the effect of MA-T *in vivo* or determining the conditions for MA-T administration under clinical conditions, attention should be paid to the appearance of cellular senescence associated with MA-T administration.

Addressing the *in vivo* toxicology aspect, a study by Noguchi *et al.* revealed that mice exposed to drinking water containing 0–3,000 μg/mL MA-T for 7 days did not exhibit adverse effects (Noguchi et al., 2022b). Furthermore, a human study involving oral care with MA-T gel containing 100 ppm MA-T for 1 month in individuals requiring nursing care for tube feeding showed a reduction in the risk of aspiration pneumonia, with no reported side effects (Ono-Minagi et al., 2023b). Given these preliminary toxicological findings, MA-T shows promise for therapeutic applications, although additional in-depth toxicological assessments are essential for its broader clinical use.

In conclusion, this study demonstrates for the first time *in vitro* that MA-T modifies sulfated residues of HSPG on odontoblasts and subsequently promotes odontoblast differentiation by activating canonical Wnt signaling without cytotoxic effects. A reanalysis of publicly available scRNA-seq data confirmed a significant enrichment of Wnt signaling activity in the odontoblast population. Wnt ligands Wnt10a and Wnt6 and Wnt inhibitors Notum and Dkk1 were identified as Wnt-related genes co-expressed in cells expressing *Dspp* and *Dmp1.* Gene silencing of *Wnt10a* and *Wnt6* synergistically downregulated *Dspp* and *Dmp1* expression, suggesting that Wnt10a and Wnt6 may be redundant in dentinogenesis. Treatment with MA-T and LiCl upregulated the expression of all the above Wnt ligands and inhibitor genes, and public ChIP-seq data indicated that all these genes had binding sites for TCF/LEF, suggesting that their expression would be autonomously regulated by a feedback mechanism. This study provided a novel approach to activate dentinogenesis by modifying HSPG and elucidated the therapeutic mechanisms of MA-T on odontoblast differentiation, suggesting the great potential application in pharmaceutical dentin regeneration. In the future, *in vivo* application of MA-T and verification of its usefulness in large animals are expected. This research is expected to lead to the application of MA-T as a more efficient pulp lining material in the future, even when dentin is lost due to caries.

## Materials and methods

### MTT assay

3-(4,5-Dimethyl-2-thiazolyl)-2,5-diphenyltetrazolium bromide (MTT) is the reagent for an assay of the metabolic activity of viable cells. The MTT assay is performed according to the manufacturer’s instructions (Sigma-Aldrich, United States). Briefly, 10 μL of MTT solution is added to each well of a 96-well plate. Incubate for 2 h in an incubator. Add 100 μL of formazan crystal solubilizer to the medium. Optical density was then measured using a microplate reader at a wavelength of 570 nm.

### Cell culture

Primary mouse dental papilla mesenchymal cells (mDP cells) were used in this study. The first molars of the mandibles of 3-day-old C57BL/6 mice were isolated with forceps under a stereomicroscope and digested for 1 h at 37 C in a solution containing 3 mg/mL collagenase type I (Sigma-Aldrich, United States) and 4 mg/mL dispase (Worthington Biochem, United States) as previously described (Wang F. et al., 2013). The enzymatically digested tissues were then filtered through a cell strainer (40 μm) to remove clumps and debris. The remaining cells were grown in Dulbecco’s modified Eagle’s medium (DMEM) (Invitrogen, United States) containing 10% fetal calf serum plus penicillin (100 units/mL) and streptomycin (100 μg/mL) at 37 C in a humidified atmosphere of air containing 5% CO_2_. Then, mDP cells were isolated by the side population (SP) discrimination assay based on efflux of the fluorescent dye Hoechst 33342 detected by FACS. Cells were maintained as a standard procedure at density ∼0.8–1.0×10^5^ cells/cm^2^ with DMEM containing 10% fetal calf serum plus penicillin (100 units/mL) and streptomycin (100 μg/mL) at 37 C in a humidified atmosphere of air containing 5% CO_2_. The medium was refreshed every 2 days, and the cells were plated after reaching confluence. The mDP cells expressed mesenchymal stem cell marker and exhibited differentiate capacity into odontoblasts, chondrocytes, and adipocytes (Supplementary Figure S4). The antibody used for immunophenotyping were purchased from BioLegend (United States, San Diego) as follows: APC-conjugated anti-CD105 (cat.120413), anti-CD73 (cat.127209), anti-CD29 (Cat.102215), anti-CD90 (cat. 140311) or APC-conjugated Isotype control IgG were used for immunolabeling of positive markers. PerCP-conjugated anti-CD45 (cat.103129), anti-CD34 (cat.119327) and PerCP-conjugated Isotype control IgG were used for immunolabeling of negative markers.

MA-T is a stable and mild chlorine dioxide-generating reagent. MA-T contains sodium chlorite, in combination with one of two kinds of cationic detergents that serve as the Lewis acid catalyzing the generation of chlorine dioxide (the Lewis acidity of both were (α: LUMO (Lowest Unoccupied Molecular Orbital) = −4.12 eV) and (γ: LUMO = −4.02)), in a buffer stabilizing the solution at a neutral pH. MA-T was provided by Acenet Inc. (Tokyo, Japan).

### Immunohistochemistry

Immunohistochemistry was performed on frozen sections of developing tooth germs and mDP cells. The sulfated HSPG distribution was visualized with 10E4 antibody (1:100; Seikagaku, Tokyo, Japan), as described previously (Hayano et al., 2012).

The immunofluorescence image was visualized using an all-in-one fluorescence microscope (BZ-X700, Keyence, Osaka, Japan) that is equipped with filters for GFP (excitation: 475 nm, emission: 525 nm) and DAPI (excitation: 360 nm, emission: 460 nm) channels. The instrument was controlled by the BZ Viewer version 1.0 software (Keyence, Osaka, Japan).

### qPCR analysis

The mDP cells were plated in 6-well plates in Dulbecco’s modified Eagle’s medium (DMEM) supplemented with 10% fetal bovine serum (FBS) until they reached confluence. The mDP cells were then cultured without or with MA-T solution at 1.0 ppm or 0.2 ppm for 48 h, or 2 weeks before processing for qPCR analysis.

qPCR was performed in triplicate on three independent sets of samples as previously described (Hayano et al., 2012; Ishimoto et al., 2015). The relative amount of transcripts was determined using a standard curve and normalized compared to the expression of glyceraldehyde-3-phosphate dehydrogenase gene (Gapdh) mRNA. The sets of synthetic primers used for the amplification were as follows: Gapdh, 5′-GTC​CCG​TAG​ACA​AAA​TGG​TG-3' (sense) and 5′-CAA​TGA​AGG​GGT​CGT​TGA​TG-3' (antisense); Mouse Dspp, 5′-AAC​TCT​GTG​GCT​GTG​CCT​CT-3' (sense) and 5′-TAT​TGA​CTC​GGA​GCC​ATT​CC-3' (antisense); Mouse *Dmp1,* 5′-CAG​TGA​GGA​TGA​GGC​AGA​CA-3' (sense) and 5′-TCG​ATC​GCT​CCT​GGT​ACT​CT-3' (antisense); Mouse *Wnt5a,* 5′-CGC​TAG​AGA​AAG​GGA​ACG​AAT​C-3' (sense) and 5′-TTA​CAG​GCT​ACA​TCT​GCC​AGG​TT-3' (antisense); Mouse *Wnt6,* 5′-TTC​CAG​TTC​CGT​TTC​CGA​CG-3' (sense) and 5′-CTG​TCT​CTC​GGA​TGT​CCT​GC-3' (antisense); Mouse *Wnt10a,* 5′-CAT​CTT​CAG​CCG​AGG​TTT​TCG-3' (sense) and 5′-AGC​CTT​CAG​TTT​ACC​CAG​AGC-3' (antisense); Mouse *Notum*, 5′-CAA​CCG​GGA​GAA​CTG​TGA​TT-3' (sense) and 5′-ACC​ACC​TCC​TGG​ATG​ATG​AG-3' (antisense); Mouse *Dkk1,* 5′-TCA​ATT​CCA​ACG​CGA​TCA​AGA-3' (sense) and 5′-GGC​TGG​TAG​TTG​TCA​AGA​GTC​TGG-3' (antisense).

Each amplification reaction was performed and checked for the absence of nonspecific PCR products by melting curve analysis using the LightCycler™ system (Roche, Germany). Relative cDNA copy numbers were calculated using data obtained from serial dilutions of representative samples for each target gene. Comparison of quantitative variables in two groups was performed using the Mann-Whitney *U* test. *p* values less than 0.05 were considered significant. Significance values were calculated using statistical analysis software with GraphPad Prism 8 (Graph Pad Software, United States).

### RNA silencing

The day before transfection, the mDP cells were plated and seeded at 150,000–300,000 cells in 35-mm cell culture dishes. Medium was replaced immediately before transfection. For transfection (all volumes per dish), 4 μL Lipofectamine RNAiMAX (Life Technologies, United States) was combined with 250 μL Opti-MEM (Life Technologies, United States) and separately 30 pmol of RNAi construct (Ambion Silencer Select Pre-designed siRNAs, Wnt10a ID:s76061; Wnt6 ID:s76095 and Universal Negative Control Number 1, Life Technologies) was used as negative control siRNA) were combined with 250 μL Opti-MEM. Both solutions were combined after 5 min and incubated for a further 20–30 min at room temperature before the transfection complexes were added dropwise to the cells. All transfections were performed in triplicate. The transfected cells were incubated for 4–6 h at 37°C in 5% CO_2_, and the medium was replaced with fresh DMEM, Ham’s F12, 10% FBS, 2 mM glutamine. Western blotting was performed after 48 h unless otherwise noted. For growth assays, transfected cells were detached by trypsinization and seeded at 4,000–5,000 cells/well.

### Mouse incisor organ culture

Mouse incisor organ culture has been previously described previously (Sarper et al., 2018). Briefly, the lower incisor was dissected from the E16.0 embryo and cultured on a track-etched polycarbonate membrane filter (Nuclepore) in Trowell-type organ culture with serum-free, chemically-defined medium (BGJB, Gibco) with or without MA-T (0.2 ppm). Tissues were harvested after 10 days of culture and processed for micro-CT and histologic evaluation.

### Micro-computed tomography (micro-CT) analysis

Cultured mouse incisors were fixed in 4% paraformaldehyde overnight and scanned by micro-CT (R_mCT2, Rigaku) at 90KV, 200 μA, microfocus 5 µm/voxel size. Volume Graphics (VGstudio) MAX 2.2 software was used to reconstruct the three-dimensional images.

### Dentin thickness quantification

Sagittal and frontal sections of mandibular micro-CT images were utilized to measure dentin thickness. Dentin thickness was measured on a micro-CT image of the frontal section at the central position in the sagittal section. From the frontal cross-sectional image of the incisor, a line was drawn through the center of the enamel shape. Dentin thickness was then measured at the lingual and labial sides along this line using ImageJ. Thresholds for identifying enamel and dentin on the labial side were determined based on segmented objects with and without dentin. The same intensity thresholds were applied to all specimens.

### Histologic evaluation

After micro-CT scanning, the fixed mouse incisors were soaked in a mild decalcifier, Osteosoft (Sigma-Aldrich), for tooth decalcification. Sagittal sections of paraffin-embedded mandibles were prepared and used for hematoxylin-eosin (HE).

### Measurement of cell viability and proliferation

Cell viability was determined using the 3-(4, 5-dimethylthiazol-2-yl)-2,5-diphenyl tetrazolium bromide (MTT) dye. Odontoblast cells were incubated with a series of different concentrations of MTT or sodium chlorite. After incubation, 10 μL MTT (Sigma-Aldrich, Japan) was added to each well of a 96-well microplate, and the microplates were placed in an incubator at 37°C for 4 h. One hundred and 50 μL of dimethyl sulfoxide (DMSO) was added to all wells and thoroughly mixed to lyse the cells and dissolve the dark blue crystals. After 10 min, the absorbance was measured at 570 nm using a microplate reader (Bio-Rad, Japan).

### Reanalysis of public scRNA-seq data

The public scRNA-seq dataset for GSE146855 of mouse incisors was downloaded from the GEO database to reanalyze the expression profile in odontoblast differentiation. The data were analyzed using a package from Seurat (version: 4.0.5) with R studio (Butler et al., 2018). In total, 6,260 cells were reanalyzed. After normalization, scaling, and principal component analysis (PCA) of the data, cells were clustered using FindNeighbors (dims = 1:6) followed by FindClusters (resolution = 0.35). The RunUMAP function was used to visualize the cell clusters.

Differential expression and cell identification were performed using FindAllMarkers (min.pct = 0.25, logfc. threshold = 0.25) with the Wilcoxon rank sum test. Visualization of gene expression by feature plot and dot plot was performed using the Seurat function FeaturePlot and DotPlot, respectively. KEGG enrichment analysis was performed on differentially expressed genes using DAVID online tools (https://string-db.org/).

### Reanalysis of public ChIP-Seq data

The bioinformatics *in silico* ChIP analysis was performed using ChIP-Atlas (https://chip-atlas.org), an integrative and comprehensive data mining suite of public ChIP-Seq data. BigWig data of Lef1, Tcf3, Tcf4, Tcf7 and RNA poly ChIP-Seq performed in different cell types were visualized around the Wnt6, Wnt10a, Notum and Dkk1 TSS. In addition, all data were mapped to the reference human genome (mouse 9) using the Integrative Genomics Viewer (IGV). Our analyses included the following publicly available ChIP atlas data sets SRX9641211, SRX1411163, SRX360319, SRX5825990.

To identify binding sites for key regulators, we searched collections in the ChIP Atlas database (https://chip-atlas.org/). This database is comprehensive and integrative, allowing visualization of public ChIP-seq data from over 131,000 experiments submitted to the Sequence Read Archives at NCBI, DDBJ, or ENA (Oki et al., 2018). The antigen class was set to ‘TFs and others’, the cell type class to ‘gonad’ and ‘embryo’, and the threshold for statistical significance values calculated by MACS2 (−10*Log10 [MACS2 Q-value]) was 20, 200, 500.

### Statistical analysis

Statistical methods were not used to predetermine sample size. Statistical analyses were performed with GraphPad Prism 8. Student’s two-sided *t*-test and two-way ANOVA were used under the assumption of normal distribution and observance of similar variance. A *p*-value of <0.05 was considered significant. Bonferroni *post hoc* analysis was performed where applicable. Values are expressed as mean ± SD. Data shown are representative images; each analysis was performed on at least three mice per genotype. Immunostaining was performed at least in triplicate.

## Data Availability

The datasets presented in this study can be found in online repositories. The names of the repository/repositories and accession number(s) can be found in the article/Supplementary Material.
