# Supplementary material for: On-demand chlorine dioxide solution enhances odontoblast differentiation through desulfation of cell surface heparan sulfate proteoglycan and subsequent activation of canonical Wnt signaling
Source: Front Cell Dev Biol. 2023 Oct 26;11:1271455. doi: 10.3389/fcell.2023.1271455 (PMC10637356; doi:10.3389/fcell.2023.1271455)
Supplement: Supplementary file 1 [file Image4.pdf]

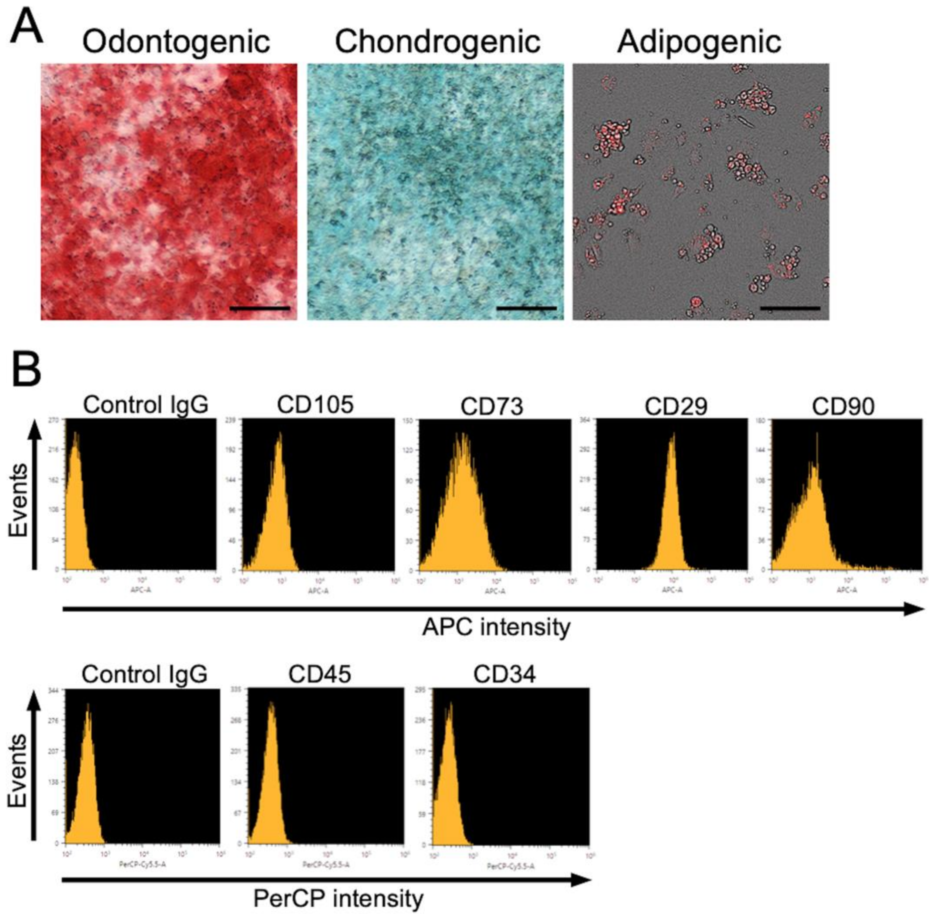

Supplemental Figure 4. Characterization of mDP cells.

(A) The mDP cells were cultured with osteogenic medium, chondrogenic medium and adipogenic medium, respectively. After 2 weeks of culture, cells were fixed and stained with alizarin red, alucian blue and lipid probe (Lipi-red), respectively. (B) The mDP cells were immunophenotyped by stem cell marker antibody and isotype controls. APC-conjugated anti-CD105, anti-CD73, anti-CD29, anti-CD90 or anti-mouse IgG were used for immunolabeling of positive markers. PerCP-conjugated anti-CD45, anti-CD34 and anti-mouse IgG were used for immunolabeling of negative markers. The cells were incubated with 1/200 diluted antibodies at 4°C for 30 min and washed. Analysis was performed by SH800ZFP (Sony). Scal bars: 200 µm in A.
