# Supplementary material for: On-demand chlorine dioxide solution enhances odontoblast differentiation through desulfation of cell surface heparan sulfate proteoglycan and subsequent activation of canonical Wnt signaling
Source: Front Cell Dev Biol. 2023 Oct 26;11:1271455. doi: 10.3389/fcell.2023.1271455 (PMC10637356; doi:10.3389/fcell.2023.1271455)
Supplement: Supplementary file 2 [file Image2.pdf]

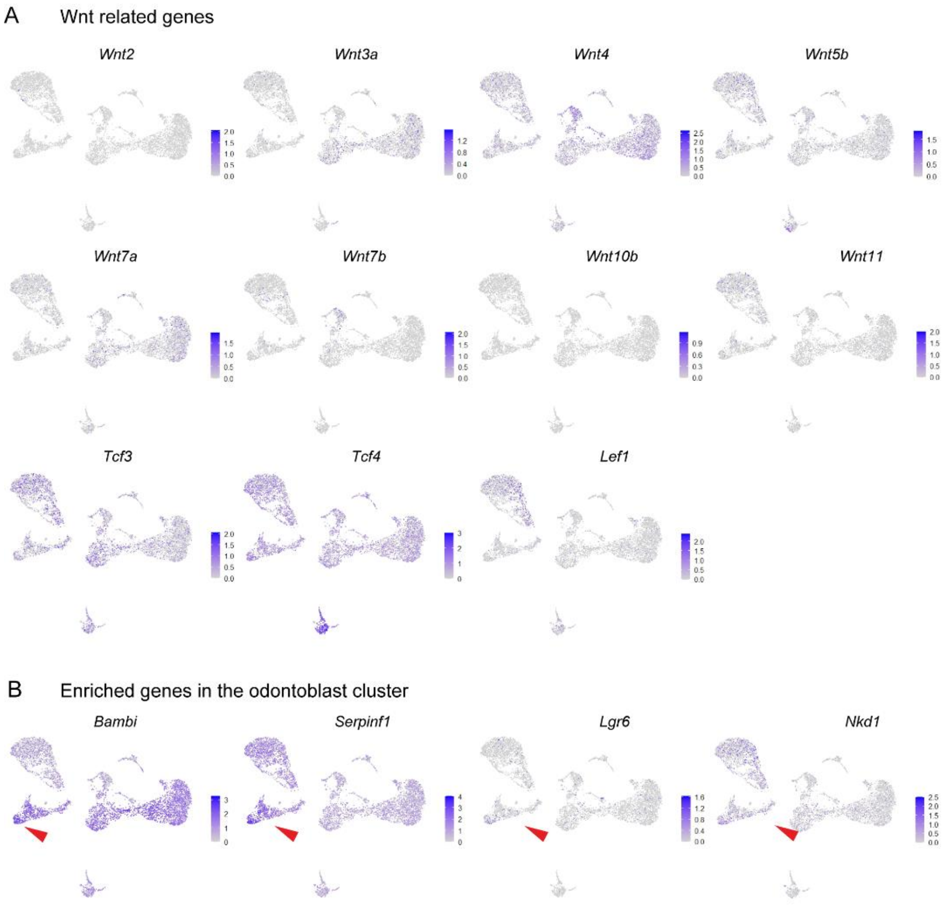

Supplemental Figure 2. Gene expression profile projected on UMAP plot.

(A) Expression of Wnt related genes projected onto UMAP plot, which did not colocalized with *Dspp* or *Dmp1*. (B) Expression of the enriched genes in "Odontoblast cluster" projected onto UMAP plot, which did not colocalized with *Dspp* or *Dmp1*.
