# Supplementary material for: On-demand chlorine dioxide solution enhances odontoblast differentiation through desulfation of cell surface heparan sulfate proteoglycan and subsequent activation of canonical Wnt signaling
Source: Front Cell Dev Biol. 2023 Oct 26;11:1271455. doi: 10.3389/fcell.2023.1271455 (PMC10637356; doi:10.3389/fcell.2023.1271455)
Supplement: Supplementary file 3 [file Image3.pdf]

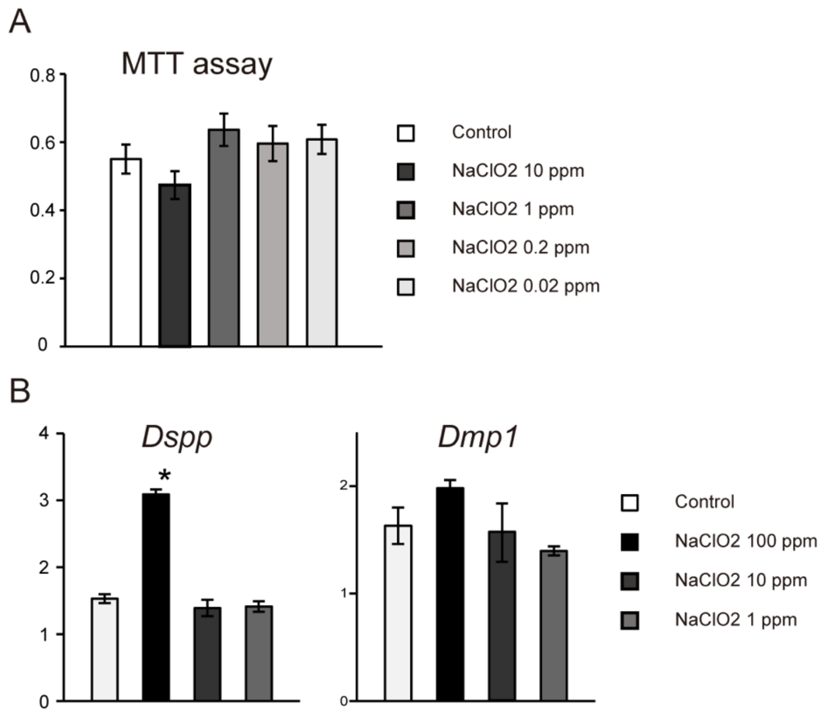

Supplemental Figure 3. Cell viability and induction of odontoblast differentiation by sodium chlorite.

(A) MTT assay for cellular viability after the exposure to 0.02 to 10 ppm sodium chlorite. MA-T exposure less than 1.0 ppm did not affect the cell viability in contrast to control. (B) A qPCR analysis of the odontoblast after treatment of sodium chlorite at different dose. sodium chlorite at 100 ppm upregulated the expression of Dspp significantly, while sodium chlorite below 10 ppm did not. *P* values were determined by one way ANOVA. *$P < 0.01$.
