# Supplementary material for: On-demand chlorine dioxide solution enhances odontoblast differentiation through desulfation of cell surface heparan sulfate proteoglycan and subsequent activation of canonical Wnt signaling
Source: Front Cell Dev Biol. 2023 Oct 26;11:1271455. doi: 10.3389/fcell.2023.1271455 (PMC10637356; doi:10.3389/fcell.2023.1271455)
Supplement: Supplementary file 4 [file Image1.pdf]

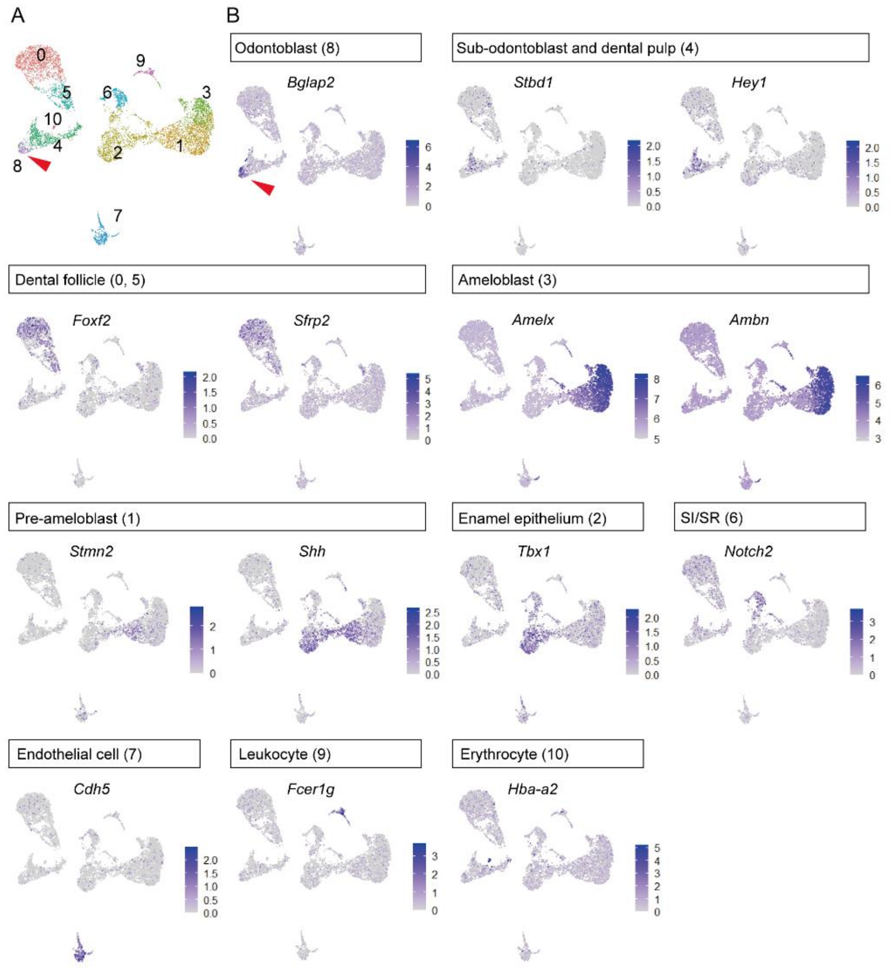

Supplemental Figure 1. UMAP analysis and identification of specific cell clusters.

(A) UMAP analysis identified ten cell population clusters including odontoblast cluster. (B) The Odontoblast cluster (#8) was characterized by *Bglp2*, and the sub-odontoblast and dental pulp cluster (#4) by *Stpd1* and *Hey1*. Dental follicle cluster (#0 and #5) were characterized by *Foxf2* and *Sfrp2*. The ameloblast cluster (#3) was marked by *Amelx* and *Ambn*, and pre-ameloblast cluster by *Stmn2* and *Shh*. The enamel epithelium cluster (#2) and stratum intermedium and stellate reticulum cluster (SI/SR, #6) were characterized by *Tbx1* and *Notch2*, respectively. Other clusters of endothelial cells (#7), leukocytes (#9) and erythrocytes (#10) were characterized by conventional markers. Expression of special markers' expression in each cluster projected on UMAP plot.
